# The National Medical Union (Official)

**Published:** 1914-04-11

**Authors:** 


### THE NATIONAL MEDICAL UNION (Official).

Offices: 346 Strand. Tel.: City 8444.

### RESOLUTIONS ON POLICY.

### The Resolutions.

At the meeting of the Council on Saturday, March 21, partly reported in these columns last week, it was resolved:—

1. To organise the medical profession to the highest possible degree of efficiency in defence of its traditional freedom and in protection of its economic interests.

2. To render every service possible to non-panel practitioners in their unequal struggle against the difficulties brought about by acceptation by panel practitioners of service under the National Insurance Acts.

3. To appoint a Parliamentary Committee, whose duties shall include lobbying, formulating questions for Parliament, approaching possible candidates at elections, and doing everything possible to bring the policy of the Union before Parliament.

4. To appoint a Committee consisting of the London members of the Council, with such other London members of the Union as may be co-opted from time to time, with power to act at its discretion in all matters appertaining to the Metropolitan area.

5. To oppose any prospective legislation to include dependants of insured persons participating in the medical benefit of the Acts under the present arrangements.

6. To agitate for drastic amendment of the National Insurance Acts in accordance with the principles of freedom on the part of the profession and public as already laid down by the Union.

7. To agitate for repeal of the Medical Benefit Sections of the Insurance Acts, or such drastic amendment as would bring these sections into harmony with the principles of freedom on the part of the profession and public as already laid down by the Union.

8. To lay down the principle of unrestricted free choice of doctor in any form of National Insurance, and to insist upon the unrestricted right of insured persons to make their own arrangements in connection with the Insurance Acts, 1911-1913; and further to insist that no doctor attending such persons be required to sign any contract with the Commissioners or any Committee under the Acts.

9. To protest against the distribution of unallotted medical benefit funds amongst panel doctors.

10. To relegate to a sub-committee for consideration and report the following principle:—

That payment of insurance premiums should be made collectively by the Insurance authority to associations of doctors, by whom the sum total should be divided on the basis of payment for work done; the systems of the National Deposit Friendly Society and the Scottish Clerks' Association to be considered and reported upon in this connection.

11. That the National Medical Union is not opposed to the principle of voluntary health insurance under such conditions as would conform with the other principles of its policy.

12. To place before the public in any and every way possible the dangers of the Acts to public health, their injustice to doctor and patient alike, and the iniquity of continuing to work them under the present conditions.

### Report of Meeting

(*Continued from* The Hospital, *April* 4, *page* 28).

In speaking to the resolution that a national medical service is necessary for the genuinely poor classes of the community, Professor Russell said that the great fault in the Act was the wage limit. If the wage limit had been made 20s. the profession would have aided the Government in working the Act. The conception of a national medical service in Edinburgh was based upon the fact that there is a large body of poor people unable to pay for medical attendance. It was the duty of the State to supply medical attendance for such people. A man with a family who has only 20s. a week could not pay for medical attendance to any great extent.

The poor, who up to the time of the passing of this Act, had been attended by charity, constituted only one-fourth of the persons who were swept into the Insurance Act, according to Lloyd George's own figures. Those were the people for whom the Government ought to have provided medical attendance, and not only for them, but for their wives and families. The twopence paid by the Government for each person in the class at present insured would have afforded a sum of eightpence a week for the genuinely poor persons, calculating them at one-fourth of the present insured persons, and this would have provided for them without taking anything from them or their employers. If the Local Government Board had really been wise enough to have done this they would have conferred a great benefit upon the community. It was upon these lines that national medical service ought really to have been formed, and as such it would have been supported by the whole profession in Edinburgh. They did not go beyond that, simply to supply the poor in this way, and to take them out of the old region of pauperism. If the National Medical Union adopted a definite policy on this point it would carry with it a great amount of moral support from the public. The public would realise that the profession was not out for spoil. Medical men would devote their lives to it perhaps as whole-time men and would obtain as much respect as men at the very top of the profession now enjoy. Make the wage limit £5 a week or 25s., but let us begin at a point where we all agree.

Mr. Wright said that the matter resolved itself into a reconstruction of the Poor Law, by which the stigma of pauperism could be obviated. He moved as an amendment that the resolution should read: "That a national medical service is necessary for the genuinely poor classes of the community and for them only." This was seconded by Dr. Burgess and carried.

**Answers to Correspondents.**

Panel Patients and Herbalists.

W. B.—We recommend you to read The Hospital of February 7, 1914, p. 20. It is immaterial whether in fact such arrangements with unqualified practitioners have, or have not, been made—the point is that the regulations issued contemplate such arrangements and provide rules to be followed in making them. We are unable to give any specific instances where State money has been paid to the unqualified under these rules, but this information you can probably obtain from the Hon. Secretaries of the National Medical Union. See also the *Sheffield Daily Telegraph*, April 4, 1914.—Ed. The Hospital.

### THE PROFESSION OF MEDICINE.

### Medical Reports at Subscribers' Annual Meetings.

As a general rule the medical staff report as presented at the average hospital to the annual meeting of subscribers and governors is non-committal even to dullness. It is usually brief, and it seldom contains any matters likely to raise controversy. Perhaps it is as well that this should be the case, for when the usual custom is departed from the results are not always satisfactory. We are prompted to offer these remarks by the reports of the speech made recently by Mr. Swinford Edwards at the annual meeting of St. Peter's Hospital for Stone and Diseases of the Urinary System, Covent Garden.

With a great deal of what Mr. Edwards, who is senior surgeon to St. Peter's Hospital, said at the meeting we are in full sympathy. If we feel obliged to criticise some passages, it is not from any want of appreciation of the fine work which is done at that hospital or of Mr. Edwards' share in doing it. Indeed, our recognition of the advances in genito-urinary surgery during the last few years and of the share which specialists (and special hospitals) have taken in them, is clearly evidenced by the comments on this subject which were published in these pages a few weeks ago (The Hospital, March 7, p. 613). On the same occasion the backwardness of some of the surgeons at general hospitals in taking advantage of modern methods was admitted. Still, the exaltation of his own institution's statistics and the simultaneous depreciation of those of other hospitals formed so prominent a feature of Mr. Edwards' speech that some protest against the principle is necessary. There can be, of course, no reasonable ground of objection to the subscribers being informed of the success of the work done by their honorary medical staff; and within limits the quoting of mortality statistics may be useful as part of such information. Comparisons, however, if they are to be instituted at all in connection with such statistics, might well be confined simply to the corresponding figures for previous years. In the case now under consideration statistics were freely quoted, apparently with the object of proving that the surgical treatment of urinary diseases is much more successful at St. Peter's than anywhere else in London. Thus statements were made which can only have been meant to show that the operation of nephrectomy has a very small mortality in St. Peter's but a very large one (more than seven times as great, in fact) in the big general hospitals. It is certain that some fallacy has crept in here; either the reports of the speech err, or the figures are inaccurate, or the statistics have been incorrectly interpreted. But whether this is so or not, comparisons of this nature are ill-advised in principle, and should be condemned by the public opinion of the institutional world.

Nor is this the only injudicious passage in the speech. In reference to the treatment of cancer of the bladder, Mr. Edwards is reported as having made certain remarks upon twelve such cases under operative treatment last year in St. Peter's. We trust he has been misreported, for he is represented as having stated that five of these twelve cases have been cured. To speak of the cure of malignant disease, wherever situated, when only a few months have elapsed since the surgical treatment is a lapse of judgment so serious that we scarcely dare suspect Mr. Edwards to have been guilty of it. It is clear, however, that he managed to convey some such impression to his audience; and since that is the case, a *caveat* is obviously necessary. The incident shows the danger of discussing too much in detail the medical and surgical work of a hospital at a meeting of lay subscribers and governors.

These criticisms apart, there was much of sound sense in the senior surgeon's speech, and it is a pleasure to acknowledge it. That the situation, construction, and equipment of St. Peter's are not ideal for a first-rate hospital we entirely agree. The appeal for a fully equipped $x$-ray department is also a matter which receives our hearty support. That St. Peter's should be able to get along at all without an $x$-ray installation is almost incredible. The absence of such a highly important—nay, essential—part of a special hospital for urinary diseases is a defect which should and must be remedied without delay. At present, we understand, the omission is remedied as far as possible by sending patients to another institution for radiography. This is all very well for cases of suspected stone, though the inconvenience of the procedure is manifest. But it must of necessity preclude altogether many desirable diagnostic aids. Thus Mr. Edwards' speech may yet serve to stimulate subscriptions, but in general terms we cannot but think that much of it was injudicious, and we hope the precedent which was set will not find many imitators.

